# Treatment and survival of Norwegian cattle after uterine prolapse

**DOI:** 10.1186/s13028-023-00701-1

**Published:** 2023-09-11

**Authors:** Adam Dunstan Martin, Per Kristian Groseth, Maien Munthe-Kaas, Ane Nødtvedt

**Affiliations:** 1 Department of Production Animal Clinical Sciences, Faculty of Veterinary Medicine, Norwegian University of Life Sciences, Postboks 5003 NMBU, Ås, 1432 Norway; 2 Present Address: Dyrehelsetjenesten i Ringsaker, Hersethøgda 239, 2355 Gaupen, Norway

**Keywords:** Bovine, Cow, Hazard, Obstetrics, Prognosis

## Abstract

**Background:**

Bovine uterine prolapse is a sporadic but life-threatening postpartum condition. The aims of this study were; (i) to determine which clinical findings determined the likelihood of treatment vs. culling, (ii) to identify the treatment methods currently employed by Norwegian veterinary surgeons and evaluate their effect on survival, (iii) to determine if clinical findings at the time of treatment could be used to determine prognosis. Practicing veterinary surgeons in Norway were contacted and asked to fill out a questionnaire on cases of bovine uterine prolapse they attended between February and October 2012. The questionnaires gathered data on signalment, clinical presentation, treatment, and outcome. These data were supplemented with culling data from the Norwegian Dairy and Beef Herd Recording Systems. The chi-squared test and logistic regression modelling was performed to identify likelihood of treatment and cox proportional hazard modelling was performed to identify the hazard of death after treatment.

**Results:**

Data from 126 cases of bovine uterine prolapse were collected (78 beef and 48 dairy cows). Twenty-six cows (21%) were emergency slaughtered, or underwent euthanasia, without treatment. Of the remaining 100 cases amputation of the uterus was performed once and repositioning was performed in 99 cases. Survival data were missing from 2 of the cases that had undergone treatment leaving a study sample of 97 cases (64 beef and 33 dairy cows). Multivariable logistic regression analysis of the explanatory variables showed that beef cows were more likely to be treated than dairy cows (OR = 0.32, 95% CI 0.13 to 0.81, P = 0.017) and that cows with a significantly oedematous or traumatised uterus were less likely to be treated (OR = 0.26, 95% CI 0.10 to 0.67, P = 0.006). Treatment methods amongst Norwegian practitioners were broadly similar. In a multivariable model cows general clinical state at time of treatment was positively correlated with survival (HR = 0.29, 95% CI 0.29 to 0.73, P = 0.008) and a history of a vaginal prolapse prepartum increased the hazard of death (HR = 2.31, 95% CI 1.08 to 4.95, P = 0.031) in the first 30 days after treatment of a uterine prolapse. In the first 180 days after treatment only veterinary assessment of a cows’ general clinical state was correlated with hazard of death (HR = 0.432, 95% CI 0.20 to 0.91, P = 0.046).

**Conclusions:**

This study shows that the production system and extent of uterine damage affect the likelihood of treatment, and that practitioners use similar treatment methods. A cows’ general clinical state at time of treatment was positively correlated with survival, and a history of a vaginal prolapse prepartum increased the hazard of death in the first 30 days after treatment of a uterine prolapse.

**Supplementary Information:**

The online version contains supplementary material available at 10.1186/s13028-023-00701-1.

## Background

Bovine uterine prolapse is a well-known, yet sporadically occurring postpartum condition. The incidence of the condition has been reported to be between 0.002 and 1% of calvings [1–5]. The proposed mechanism for its development is decreased myometrial tone combined with an open cervix – which explains why hypocalcaemia, and dystocia (which can result in myometrial fatigue), are risk factors for the condition [6]. It further explains why the anecdotal findings of the authors that uterine prolapse is more common in primiparous beef cows and older dairy cows, a pattern described by Murphy and Dobson [6]. In contrast to vaginal prolapse, incidence of uterine prolapse does not seem to have a clear hereditary component link, or increased likelihood of recurrence after subsequent calvings [2]. These factors combined with the sporadic nature of the disease mean that specific herd level management protocols to prevent it are unlikely to be put into place.

Uterine prolapse should be regarded as a condition which requires emergency treatment. Without timely intervention the prognosis for life is grave [2]. Whilst there is no empirical evidence that uterine prolapse is painful per se, it seems reasonable to assume the condition is associated with, at the very least, a degree of discomfort which negatively impacts on the individual’s welfare [7]. The first decision to be made on diagnosis of a uterine prolapse is as to whether the cow should be treated or be culled – either by euthanasia or on farm emergency slaughter [8]. In Norway, in contrast to many other countries, on farm emergency slaughter is a genuine option for these animals which provides a farmer the option of salvaging some of the economic value of the dam, providing certain prerequisites are met [9].

Treatment methods are well described in textbooks and scientific articles [2, 10–12]. The protocols have several similarities – clean the uterus, caudal epidural anaesthesia, lift the uterus before replacement. However, certain controversies remain, including; the use of tocolytics, oxytocin, and antimicrobials as well as whether suturing the vulva after replacement of the uterus is necessary [11]. However, there are no recent studies documenting the treatments given by veterinary surgeons in the field. The likelihood of survival after treatment has been documented to be between 61 and 83% [1, 3, 5, 13]. However, there is a paucity of published literature documenting the success of different treatments [11, 13] and even fewer papers which try to identify prognostic indicators which can be established before treatment starts [7]. The lack of evidence on which to make clinical decisions led to a review of expert opinion being published 2011 [11]. Despite the expert opinion piece illustrating the lack of evidence on which to base clinical decisions, few data on uterine prolapse in *bos taurus* cows have been published [4].

This study therefore has the following aims; (i) to determine which clinical findings determined the likelihood of corrective treatment, opposed to on farm emergency slaughter and euthanasia for uterine prolapse, (ii) to identify the treatment methods currently employed by Norwegian veterinary surgeons and evaluate their effect on survival, (iii) to determine if clinical findings at the time of treatment could be used to inform prognosis.

## Methods

This study consists of data gathered from two questionnaire-based studies which has been supplemented with data from the Norwegian Dairy Herd Recording System (NDHRS) and the Norwegian Beef Cattle Recording System (NBCRS).

### Case selection and data collection

The first questionnaire-based study ran from February until October 2012. Inclusion was determined by first identifying the 100 council municipalities in Norway with the highest cattle population using subsidy payment data from the Norwegian Agricultural Agency (www.slf.dep.no/en). Telephone calls were made to private veterinary practices believed to be working with cattle in each of these areas and veterinary surgeons were asked if they would be willing to submit data from one or more cases of bovine uterine prolapse, they attended in the study period. Questionnaires were sent to those that agreed by post and reminders were sent by email twice during the study period.

The second questionnaire was performed between February and July 2012. A convenience sample of 23 veterinary surgeons in the five counties with the highest beef cow population in Norway were recruited and asked to collect a blood sample and fill in a questionnaire when they attended a case of uterine prolapse in a beef cow. The blood sample was to be analysed without cost to the producer or veterinary surgeon for some minerals and metabolites (calcium, potassium, magnesium, selenium, urea, beta-hydroxybutyrate), the results of these samples do not appear in this study but are mentioned as this incentive may have encouraged responses.

The questionnaires sent out were similar and included questions on breed, parity, dystocia, pre-partum vaginal prolapse, the cows’ position at the time veterinary attention was provided, the time from calving until prolapse and the condition of the uterus at the time reparation commenced, and if the veterinary surgeon believed they had completely replaced the uterus. The first questionnaire also included more details on treatment provided. Data on cow survival were gathered from the national databases – the Norwegian Dairy Herd Recording System (NDHRS) and the Norwegian Beef Cattle Recording System (NBCRS). These voluntary production databases held information on majority of the beef (> 77%) and dairy (> 97%) cows in Norway in 2022 and have been used in previous research [14, 15].

### Data handling

Data from the two questionnaires were recorded in individual databases (Microsoft Office Excel; Microsoft Corporation Redmond, WA) prior to these being merged to make one complete dataset. Details on individual cow signalment and time of culling were checked against the respective national databases (NDHRS and NBCRS). Data referring to signalment, clinical presentation and treatment were categorised and tabulated. When detailed information about treatment was missing the variable in question was set as missing and the case retained for other analyses. For example, if a veterinary surgeon had not stated explicitly that oxytocin was used, or not used, the information was classified as missing and excluded from analyses regarding the use of oxytocin, but the case still appears in all other analyses. The categories and numbers in each category can be seen in Table 1 (Signalment and clinical presentation) and Table 2 (Treatment).

**Table 1:** Univariate analysis of explanatory variables and survival outcomes of 116 cases of bovine uterine prolapse

Variable	Level	Number of cases	Day 0			Number of cases with treatment and survival data	Day 30			Day 180		
			Treated	Slaughtered /euthanasied	P-value chi squared		Number of deaths 0 to 30	Mean time to death	P-value cox	Number of deaths	Mean time to death	P-value cox
**Production**	Beef	78	67	11	**0.021**	64	19	7.3	0.528	40	19.2	0.313
	Dairy	48	33	15		33	8	9.5		17	20.5	
**Parity**	Primiparous	94	79	15	0.225	76	18	7.2	0.114	45	21.0	0.782
	Multiparous	32	21	11		21	9	9.4		12	15.6	
**Hold**	< 2.75	19	12	7	0.644	13	5	11.1	0.663	10	20.6	0.417
	2.75 to 3.5	63	52	11		50	12	5.3		27	19.0	
	> 3.75	44	36	8		34	10	9.5		20	19.7	
**Clinical state**	Good/Acceptable	108	90	18	**0.007**	87	21	7.9	**0.030**	49	20.5	**0.046**
	Poor	18	10	8		10	6	7.9		8	13.4	
**Recumbent**	Yes	81	60	21	**0.049**	59	18	8.6	0.510	37	19.6	0.207
	No	45	40	5		38	9	6.7		20	19.5	
**Dystocia**	Eutocia	75	58	17	0.529	56	19	7.1	0.118	34	17.2	0.651
	Dystocia	50	42	8		40	8	9.8		23	23.0	
	Missing	1										
**Milk fever**	absent	81	63	18	0.873	61	13	8.8	0.341	33	21.7	0.600
	Present	10	2	8		8	3	5.3		5	15.2	
	Missing	35										
**Placenta attached**	no	51	40	11	0.538	39	8	3.8	0.630	22	20.5	0.905
	Yes	40	30	10		30	8	12.4		16	21.2	
	Missing	35										
**Damage to uterus**	None	69	61	8	**0.006**	58	16	6.7	0.959	35	19.3	0.740
	Traumatised and/or oedematous	57	39	18		39	11	9.8		22	19.9	
**Pre-partum vaginal prolapse**	No	81	64	17	0.052	62	49	5.4	0.053	34	20.6	0.306
	Yes	44	35	9		34	20	10.3		23	18.0	
	Missing	1										

**Table 2:** Details of treatment provided to, and survival of, 96 cases of bovine uterine prolapse

Treatment	Group	Number	Day 30		
			Deaths	Mean time to death (days)	P-value*
**Epidural**	No	18	6	5.8	0.561
	Yes	78	20	7.5	
**Calcium therapy**	No	75	20	8.3	0.854
	Yes	18	5	3.5	
**Systemic antibiotic therapy**	No	67	20	7.5	0.512
	Yes	22	5	6.4	
**Intrauterine antibiotic therapy**	No	58	20	8.6	**0.072**
	Yes	31	5	2.7	
**Oxytocin**	No	31	7	12.5	0.430
	Yes	65	19	5.1	
**Non-steroidal anti-inflammatory drugs**	No	80	23	6.6	0.370
	Yes	16	3	11.3	
**Vulval suture**	No	2	0	-	-
	Yes	94	26	7.1	
**Used prolapse board**	No	12	1	1.0	0.191
	Yes	57	14	7.2	
**Used cold water**	No	31	5	6.1	0.328
	Yes	38	10	7.1	
**Used sugar**	No	68	15	6.8	-
	Yes	1	0	-	
**Felt confident completely replaced**	No	17	4	7.3	0.840
	Yes	52	11	6.6	
**Time to replace**	≤ 0 min	46	7	7.1	**0.008**
	> 20 min	51	20	8.2	

*Calculated using cox proportional hazard analysis

### Statistical analyses

Statistical analyses were performed in Stata 15 (StataCorp., College Station, TX). In all analyses, statistical significance was defined as a P-value ≤ 0.05. A tendency was determined by a P-value ≤ 0.10. Explanatory variables which were found to have a P-value ≤ 0.20 in univariable analyses were included in multivariable models which were constructed using a backwards manual selection procedure, which included checks for collinearity.

### Association between clinical presentation and treatment

There are four broad treatment options when treating a case of uterine prolapse. These are repositioning, amputation, on farm emergency slaughter, or euthanasia. In this study the binary outcome variable ‘treated’ is reserved for those cases in which the veterinary surgeon attempted to preserve life by either trying to perform reduction or amputation of the uterus. The association between the outcome variable treated and the explanatory variables, listed in Table 1, were explored with univariate chi-squared testing. A multivariable model was built using the procedure described above. Once the final model was built it was checked for interaction between the terms using the Wald statistic and for confounding by removing variables and assessing the degree of change that occurred in each coefficient. Finally, the goodness-of-fit for the model was assessed using the Hosmer-Lemeshow test.

### Survival analyses

Survival analyses were performed, the outcome variable was time from prolapse until culling and death was the endpoint/failure. The day the prolapse was attended by a veterinary surgeon was defined as Day 0. The time to event was defined as time from examination by the veterinarian (Day 0) until time of death (which included unassisted death, euthanasia, on farm emergency slaughter and slaughter at an abattoir) or censoring because of loss to follow-up or end of study period. Time in days was generally calculated as an integer. In the situation that a treated cow died on the same day as treatment the value 0.5 was assigned to the outcome variable. Two different analyses were performed. In the first analysis the follow-up period ended at Day 30 and all lactations were right censored at this time. In the second analysis they were right censored at Day 180. The term ‘end day’ was used to describe right censoring.

Survival analyses were used to investigate both the association between clinical presentation and survival, and the association between treatment received and survival. The method of investigating both groups of associations was similar. The influence of the explanatory variables on time to event data were explored graphically using histograms, scatter plots, and Kaplan Meyer plots. Time to event data were then analysed using Cox proportional hazard modelling techniques. The start day, end day, and definition of event have been previously described. The unit of study was an individual calving. The explanatory variables were tested in univariate cox-proportional hazard analysis. A crude hazard ratio for each explanatory variable was generated. A multivariable model was then built using the procedures described above. Once complete the model was checked to confirm that the hazards were proportional over time using both the Kolmogorov-Smirnov test and the Cramer von Mises test.

## Results

Data were collected from 126 cases of bovine uterine prolapse. In total 92 cases were reported via the first questionnaire from 57 different veterinary surgeons. Whilst the second questionnaire contributed 34 cases provided by 22 veterinary surgeons. Survival data were missing from one first and one third parity beef cow and these animals were excluded from all survival analyses, but treatment methods have been reported. Of the remaining 124 cases 48 were from dairy cattle (16 primiparous, 22 multiparous) and 76 from beef cattle (43 primiparous, 33 multiparous).

In total 100 cows (79%, 67 beef cows and 33 dairy cows) were treated, and 26 animals (21%, 11 beef and 15 dairy) were immediately euthanized (n = 4) or underwent emergency slaughter (n = 22). The primary form of treatment (99 cases) was repositioning of the uterus. Amputation of the uterus was performed in one animal (seventh parity beef cow), this animal has been excluded from time to event analyses. Any form of survival data were missing from two dairy cows which have been removed from the survival analyses. Further details on the presentation of the animals, including parity, body condition score, clinical impression and concurrent diseases can be found in Table 1.

When the explanatory factors for treatment were evaluated in a multivariable logistic regression model (Table 3) cows with a significantly oedematous or traumatized uterus were less likely to be treated than those without visible injury to their uterus (OR = 0.26, 95% CI 0.10 to 0.67, P = 0.006). Additionally, beef cows were more likely to be treated than dairy cows (OR = 0.32, 95% CI 0.13 to 0.81, P = 0.017). Although dairy cows were more likely to be in a worse clinical state than beef cows (P = 0.000).

**Table 3:** Multivariable logistic regression model showing the likelihood of treatment of cows with uterine prolapse

Variable	Level	Odds ratio	Standard Error	95% confidence interval		P-value
				Lower	Upper	
Intercept		51.71	46.16	8.99	297.47	0.000
Production system	Dairy	0.32	0.15	0.13	0.81	0.017
	Beef	Baseline				
Condition of uterus	Damaged/oedematous	0.26	0.13	0.10	0.67	0.006
	Normal	Baseline				

Of the 97 treated by repositioning of the uterus for which survival data was available 27 (28%) died within 30 days of treatment. The cow which was treated by amputation was reported to be alive and well three days after the surgery, but further data on survival data were missing. Cows that were evaluated to be in a normal or slightly reduced clinical state when treated had a lower hazard of death (HR = 0.03, 95% CI 0.01 to 0.08, P = 0.030) in first 30 days and 180 days (HR = 0.43, 95% CI 0.20 to 0.91, P = 0.046) post-prolapse treatment than those who were evaluated to be in poor clinical state. A tendency was seen for cows which had suffered a vaginal prolapse during pregnancy to have a higher hazard of death in the first 30 days post-prolapse treatment than those which did not (HR = 2.12, 95% CI 0.99 to 4.51, P = 0.054). No other univariate associations were seen between the tested explanatory variables and hazard of death post-treatment. When building the multivariable model animals that had poor clinical state and previous vaginal prolapse were found to have a significantly higher hazard of death than those who did not in the first 30 days post prolapse (Table 4).

**Table 4:** Cox proportional hazards model investigating hazard of death following treatment uterine prolapse treatment (30 days)

Variable	Level	Estimate	Standard Error	95% confidence interval		P-value
				Lower	Upper	
General clinical state	Normal/slightly reduced	0.29	0.14	0.12	0.73	0.008
	Poor	Baseline				
Prepartum vaginal prolapse	Reported	2.31	0.90	1.08|	4.95	0.031
	Not reported	Baseline				

Some treatment data was available from 96 cases. To summarise, 84% of reported treatments included the use of an epidural, 24% included the use of systemic antibiotics and 35% the use of intrauterine antibiotics. Two thirds (68%) of cases received oxytocin and some form of vulval suture was used in 98% of cases. Table 2 details the treatments given their effect on survival up to Day 30 and comparison between the treatments using a univariate cox proportional hazard analysis. A tendency seen in the data for cows treated with intrauterine antibiotics to have a lower hazard of death compared to those that were not treated with inter-uterine antibiotics (HR 0.43, 95%CI 0.16 to 1.16, P = 0.072). However, there was no other significant associations seen between pharmaceutical treatments and hazard of death. In most cases (83%) treatment was facilitated by using a prolapse board, or other structure to lift the uterus. Cold water was also used 55% of cases to facilitate the reparation of the uterus. The use of aids to facilitate reparation of the uterus did not affect the hazard of death in the first 30 days of treatment.

Veterinary surgeons felt confident that they had replaced the entire uterus in 75% of cases, their feeling of successful reparation was not associated with an altered hazard of death by Day 30. However, the time taken to replace a uterus was positively associated with hazard of death. If the treatment time for repairing a uterus was more than 20 min the hazard of death was almost three times greater in the first 30 days after treatment than those which took 20 min or less to replace (HR 2.96, 95% CI 1.25 to 7.01, P = 0.008).

## Discussion

The crude survival of cows which had suffered uterine prolapse in this study was low, 58% were alive 30 days later. This is similar to the study performed by Ishii et al. in Japan (61% crude survival) [13]. However, low compared earlier to studies by Carluccio (82%) [4] and Ødegaard (74%) [1]. In the current study 21% of animals were euthanised or underwent on farm emergency slaughter before treatment commenced, which is higher than the 10% reported by Ødegaard in 1977, and the 13% reported by Ishii et al. [13]. The reason that such a high proportion of the animals suffering from the condition were euthanized may be linked to large distances between farms and veterinary care in Norway. However, the most likely explanation is that there is a functional on farm emergency slaughter system available throughout Norway, meaning that many veterinarians and farmers would choose to ‘cut their losses’ and slaughter a cow to salvage some of her value rather than risking losing all her value by treating her [8, 9]. This opinion is supported by the positive univariate correlation seen in this study between general clinical state and likelihood of treatment.

Beef cows were more likely to be treated than dairy cows. Whilst it is true that dairy cows were more likely to be in a poor clinical state than beef cows, it is likely that the decision to treat beef cows is a multifactorial decision, with the desire to keep the dam alive to suckle perhaps being a motivation. The payment system for cattle undergoing on farm emergency slaughter in Norway is such that a greater proportion of the value of a beef cow, compared to a dairy cow, is lost if it is slaughtered on farm rather than in a slaughterhouse. Statistically there was no difference in hazard of death for beef and dairy cattle after treatment, which perhaps indicates that some more dairy cows could have been treated and recovered. Both recumbent animals and those with an injured and/or oedematous uterus were less likely to be treated than standing animals with an undamaged uterus. Both factors also are likely to have contributed to the overall clinical assessment of the animal. Animals that were recumbent and/or had an injured uterus were probably more likely to be considered in a poor state than those that were standing with no visible complications This is supported by the fact that in the multivariable logistic regression model built to investigate factors associated with treatment both production system and uterine injury remained in the final multivariable model whilst general clinical appearance did not.

Most veterinary surgeons treated uterine prolapse similarly. The overwhelming majority (> 80%) used caudal epidural analgesia, and a prolapse board (or some other aid to lift the uterus prior to its replacement). The vulval lips were held together in some way in all but two of the treated cases. This is interesting as the value of suture placement has long been debated and in fact no evidence base is available to show that this practice is beneficial [2, 10, 11]. Further it is known that placement of sutures can have negative implications for an animal’s health if prolapse recurs [2, 10]. A previous study has shown that a considerable proportion of veterinary surgeons do not feel that sutures have a benefit – but place them if the farmers would like them to be [11]. Whilst this is understandable it does present some ethical dilemma given that the founding stone of medical and veterinary ethics is *primum non nocere* (“above all, do no harm”) [16]. Therefore, further study into the value of placing potentially harmful sutures should be undertaken.

The immediate, and 30-day, survival rate after treatment in this study was similar but slightly lower than the majority of [1, 3–5, 17], but not all, studies [13]. The current study looked at 30-day and 180-day survival, other studies have typically used a two-week period to investigate the immediate survival after prolapsing [3, 5, 13]. The longer time was used in this study to include animals that may have been treated with pharmaceuticals before being slaughtered once medicine withdrawal periods were complete. Longer term survival in a herd is complicated and often linked to reproductive performance and herd management issues. The reproductive performance of these animals was not studied in the current study because the study design did not lend itself to gathering reliable information on these events.

Only two factors were found to significantly influence survival in the first 30 days post treatment. These were general clinical state as assessed by the attending veterinary surgeon and the time taken for a veterinary surgeon to reposition the uterus. If reparation took 20 min or less hazard of death in the first 30 days was lower than if reparation tool more than 20 min. It is important to be aware that the effect described is an association which is not the same as a causal relationship. Ideally prognostic indicators would allow for a veterinary surgeon to decide whether to commence treatment or not. However, knowledge of post-treatment prognosis is important as has been highlighted by Gregory [7], who also found a correlation with survival and rapid uterine reparation. Most practitioners did not use antimicrobial treatment routinely when replacing a uterus, which contrasts with expert opinion on the subject [11]. This may be because they felt this was not necessary or it may be because of a desire to not prohibit the on farm emergency slaughter of animals by treating with medicines that have a meat withdrawal period. It is therefore interesting that the use of local intrauterine antibiotics tended to be associated with a lower hazard of death in the 30 days post treatment in this study. This effect was not seen in the multivariable model. It should be noted that this study was not designed compare treatments and define a gold standard treatment so these findings must be treated with caution.

The value of veterinary opinion regarding general clinical appearance was shown in the current study to be correlated with the hazard of death after treatment up until Day 30 and Day 180 in the univariate survival analysis. This is an important finding as it demonstrates that veterinary clinical opinion is important – and the most accurate prognostic indicator determined by this study. The method used by veterinary surgeons to evaluate clinical state was left ‘open’. In other words, no guidance as to which criteria should be evaluated were included in the questionnaire. Discovering more about how veterinarians evaluated clinical state could be an interesting area for further research.

The strength of the association was such that general clinical appearance also remained in the final multivariable survival model which was built to see which factors impacted on the hazard of death in the first 30 days after treatment. Interestingly the occurrence of vaginal prolapse in the pregnancy prior to the prolapse also tended to be associated with an increased hazard of death in the 30 days following treatment of uterine prolapse. This is the first study reporting this relationship. It makes biological sense, as a cow that has previously suffered from a vaginal prolapse may be more likely to have a pre-existing pathology in the reproductive tract at the time of uterine prolapse which could potentially provide irritation making a recurrence of the prolapse more likely. Damaged vulval or cervical tissue may also be infected by bacteria which could increase the likelihood [2] of infection of the uterus with pathogenic bacteria leading to post-partum uterine infection and increased mortality.

A positive association between the incidence of vaginal prolapse and uterine prolapse has been previously reported in Norway in the 1970’s [1]. However, work performed worldwide after this has not described this relationship [2]. Vaginal prolapse in pregnant cows is a common finding which is generally only reported if the prolapse is severe and confers a problem for the cow. Consequently, it is likely that the true incidence of vaginal prolapse is under-reported. A possible association between vaginal prolapse, uterine prolapse and survival after a uterine prolapse could potentially be linked but under-reporting has meant that this association has been difficult to document in international studies. It could also be that there is a predisposition for this relationship in Norway – where the predominant dairy breed is the Norwegian Red. This article highlights the need for more knowledge in this area.

Unfortunately, the statistical power to identify differences between the explanatory variables in this study is low. Ideally the population size would have been greater. Selection of veterinary surgeons to participate in this study was not random and some data collection was incentivised (blood sampling). Previous researchers have commented that the sporadic nature of uterine prolapse has made the data collection difficult. It is unlikely that the sampling will have biased the results of the study. Not targeting participation would have likely reduced the number of cases reported and reduced the statistical power of the study. Despite having limited statistical power, this study has value as it not only revisits an old problem 30 years after most of the research in the field was published, but also because it allows for other researchers to use these data in combination with reports from other workers to perform a meta-analysis of data to provide a stronger evidence base on which to make clinical decisions. These data are limited in so much as they refer to Norwegian cattle and Norwegian production systems. Those looking to apply the findings of this study elsewhere should consider the presence of a well-functioning on farm emergency slaughter service and the fact that predominant dairy breed in Norway is the Norwegian Red when interpreting data. However, within Norway the study has a high validity and is directly applicable to farm animal practice.

## Conclusions

This study found that production system (dairy less likely than beef) and uterine damage affected the likelihood of treatment after a uterine prolapse in cows. Treatment methods amongst Norwegian practitioners were broadly similar. A cows’ general clinical state at time of treatment was positively correlated with survival and a history of a vaginal prolapse prepartum increased the hazard of death in the first 30 days after treatment of a uterine prolapse.

### Electronic supplementary material

Below is the link to the electronic supplementary material.

Supplementary Material 1

## Data Availability

The datasets used and/or analyzed during the current study are available from the corresponding author on reasonable request.

## Declarations

### Prior publication

Data have not been published previously.
