# Supplementary material for: Treatment and survival of Norwegian cattle after uterine prolapse
Source: Acta Vet Scand. 2023 Sep 11;65:38. doi: 10.1186/s13028-023-00701-1 (PMC10496322; doi:10.1186/s13028-023-00701-1)
Supplement: Supplementary file 1 — Supplementary Material 1 [file 13028_2023_701_MOESM1_ESM.docx]

**Questionnaire on Bovine Uterine Prolapse**

**General**

Date:

Veterinary Surgeon:

Animal Owner and Producer Number:

Type of Housing (cross as appropriate):

🞏 Cubicle House, 🞏 Straw yard, 🞏 Tie stalls, 🞏 Other, please describe…………………………………………

**Animal Details**

Cow identification number:

Breed:

Year of birth:

Parity:

Body Condition Score (1 to 5 scale 1=thin, 5=obese) include decimal point:

**Clinical evaluation**

Cow’s general clinical state (given uterine prolapse): 🞏 Poor, 🞏 Typical, 🞏 Good.

On arrival could the cow stand: 🞏 Yes 🞏 No.

Prior to calving had the cow experienced vaginal prolapse:

Estimated time from calving until treatment:

Dystocia: 🞏 None, 🞏 Slight, 🞏 Considerable

Condition of the uterus when prolapsed (tick all that apply): 🞏 Normal, 🞏 Odematous, 🞏 slight damage, 🞏 Considerable damage, 🞏 Other, please describe ………………………………

Was the placenta attached to the uterus?: 🞏 Yes, 🞏 No.

Did the cow have clinical milk fever?: 🞏 Yes, 🞏 No.

Do you have any other significant clinical observations to make: ………………………………………………….

**Treatment**

What was the primary form of treatment: 🞏 Repositioning, 🞏Amputation, 🞏Emergency Slaughter, 🞏 Euthanasia.

Answer the following questions as appropriate:

How long did the repositioning of the uterus take: 🞏 0-20 minutes, 🞏20-40 minutes, 🞏 40-60 minutes, 🞏 >60 minutes.

Are you confident both horns were completely replaced: 🞏 Yes, 🞏 No, 🞏 Unclear.

Did you use any aids to help reposition the uterus: 🞏 Bottle or similar, 🞏 Sugar, 🞏 Cold water, 🞏 Prolapse board, 🞏 None were used, 🞏 Other, please describe……………………………………………..

Did you place a vulval suture: 🞏 Yes, 🞏 No.

If so which vulval suture did you use: 🞏 Subcutaneous Bühner suture, 🞏 Mattress suture, 🞏Flessa suture, 🞏 Other, please describe……………………

Was an epidural used?: 🞏 Yes, 🞏 No. If yes name which drug was used?

Was oxytocin administered?: 🞏 Yes before repositioning, 🞏 Yes after repositioning, 🞏 No.

Was calcium administered?: 🞏 Yes before repositioning, 🞏 Yes after repositioning, 🞏 No.

If Calcium was administered how was it administered?: 🞏 intravenous, 🞏 subcutaneous, 🞏 orally.

Were intrauterine antibiotics used?: 🞏 Yes, 🞏 No. If yes name which drug was used?

Were systemic antibiotics administered?: 🞏 Yes, 🞏 No. If yes name which drug was used?

Were non-steroidal anti-inflammatory drugs administered?: 🞏 Yes, 🞏 No. If yes name which drug was used?

Do you have an other relevant comments regarding the case, including other treatment methods used or drugs administered?
